# Recent Advancements in Metal–Organic Framework-Based Microfluidic Chips for Biomedical Applications

**DOI:** 10.3390/mi16070736

**Published:** 2025-06-24

**Authors:** Alemayehu Kidanemariam, Sungbo Cho

**Affiliations:** 1 Department of Electronic Engineering, Gachon University, Seongnam-si 13120, Gyeonggi-do, Republic of Korea; alemayehukim@gachon.ac.kr; 2 Department of Semiconductor Engineering, Gachon University, Seongnam-si 13120, Gyeonggi-do, Republic of Korea; 3 Gachon Advanced Institute for Health Science & Technology, Gachon University, Incheon 21999, Republic of Korea

**Keywords:** MOF-based nanozymes, microfluidic chips, biomedical application, microfabrication, cell migration, point-of-care diagnostics

## Abstract

The integration of metal–organic frameworks (MOFs) with microfluidic technologies has opened new frontiers in biomedical diagnostics and therapeutics. Microfluidic chips offer precise fluid control, low reagent use, and high-throughput capabilities features further enhanced by MOFs’ ample surface area, adjustable porosity, and catalytic activity. Together, they form powerful lab-on-a-chip platforms for sensitive biosensing, drug delivery, tissue engineering, and microbial detection. This review highlights recent advances in MOF-based microfluidic systems, focusing on material innovations, fabrication methods, and diagnostic applications. Particular emphasis is placed on MOF nanozymes, which enhance biochemical reactions for multiplexed testing and rapid pathogen identification. Challenges such as stability, biocompatibility, and manufacturing scalability are addressed, along with emerging trends like responsive MOFs, AI-assisted design, and clinical translation strategies. By bridging MOF chemistry and microfluidic engineering, these systems hold great promise for next-generation biomedical technologies.

## 1. Introduction

Microfluidic technologies have transformed the landscape of biomedical research and diagnostics over the past two decades by enabling the precise manipulation of fluids at the microscale (typically from tens to hundreds of micrometers) [1,2]. These lab-on-a-chip systems offer significant advantages over conventional techniques, including reduced reagent consumption, faster analysis, portability, and real-time monitoring capabilities [3]. Their integration with diverse detection modalities such as electrochemical, optical, and magnetic methods has positioned microfluidics as a core technology in applications ranging from pathogen identification to cancer diagnostics and personalized medicine [4,5].

However, despite these advances, conventional microfluidic platforms often face challenges related to sensitivity, selectivity, and multifunctionality, which limit their broader biomedical applications and necessitate the incorporation of advanced functional materials to enhance device performance [6].

Metal–organic frameworks (MOFs) have recently emerged as a groundbreaking class of crystalline porous materials composed of metal ions or clusters coordinated to organic ligands, forming highly ordered three-dimensional networks [7]. This modular structure endows MOFs with exceptional properties such as ultrahigh surface areas, tunable pore sizes, and versatile chemical functionalities that can be tailored at the molecular level [8]. These features make MOFs particularly well suited for biomedical applications including selective molecular recognition, catalysis, drug encapsulation, and controlled release [9]. In addition, certain MOFs exhibit enzyme-mimicking catalytic activities known as nanozyme behavior that offer enhanced stability and cost-effectiveness compared to natural enzymes [10]. The combination of these attributes has positioned MOFs as promising materials for biosensing, bioimaging, antimicrobial therapy, and targeted drug delivery.

The integration of MOFs with microfluidic systems marks a significant advancement in biomedical device engineering. This combination enables the development of high-performance platforms that unite the precision and scalability of microfluidics with the multifunctionality of MOFs [11]. MOF-enhanced microfluidic chips demonstrate improved detection sensitivity, specificity, and versatility, capable of capturing and analyzing biomolecules like DNA, proteins, and pathogens through various signal transduction mechanisms, including electrochemical, fluorescent, and colorimetric outputs [12,13]. Furthermore, MOFs can be spatially engineered within microchannels to create specialized reaction zones for multiplexed assays, facilitating high-throughput screening and complex biological simulations [14,15].

Recent progress in fabrication techniques such as in situ MOF growth, layer-by-layer assembly, and 3D printing has further expanded the design possibilities for MOF-microfluidic platforms. These systems are increasingly being applied to point-of-care diagnostics, organ-on-a-chip models, and therapeutic monitoring [16,17]. In drug delivery, MOFs offer controlled release and targeting capabilities; in wound healing, they enable antimicrobial action and tissue regeneration; and in diagnostics, they improve sensitivity and detection speed [18,19,20,21]. Continued efforts in optimizing biocompatibility, biodegradability, and large-scale manufacturing are accelerating their path toward clinical translation.

Given the rapid evolution of this interdisciplinary field, this review provides a comprehensive overview of recent advancements in MOF-based microfluidic chips for biomedical applications. It explores the principles of material design, integration strategies, and emerging applications, while critically addressing current limitations and highlighting future directions including smart MOFs, AI-assisted design, and regulatory considerations for commercialization. By bridging the gap between MOF chemistry and microfluidic engineering, this work aims to inspire innovation in next-generation biomedical technologies.

## 2. Biomedical Applications of MOFs: From Material Innovations to Microfluidic Devices

Metal–organic frameworks (MOFs) offer unique advantages for biomedical applications due to their high surface area, tunable porosity, and structural flexibility. These characteristics facilitate effective drug encapsulation, regulated release, and surface modification for targeted therapeutic delivery [22]. MOFs have also shown promise in biosensing and imaging, particularly when designed to respond to physiological stimuli [23]. Advances in biocompatibility and degradability have further enhanced their potential for safe and effective use in clinical settings.

### 2.1. Drug Delivery and Controlled Release

MOFs have gained attention as adaptable drug delivery carriers owing to their substantial loading capacity, customizable pore dimensions, and protective environment that shields therapeutic agents from degradation [14]. Their surfaces can be functionalized for targeted delivery, while their porous structures enable controlled and sustained drug release. For example, Tran eta al. developed ZIF8 efficiently loaded doxorubicin (62 mg/g) and, when functionalized with polyacrylic acid (PAA), it exhibited good dispersibility, fluorescent imaging, and pH-responsive release [24]. In vitro, it released 24.7% at pH 7.4 and 84.7% at pH 4.0, demonstrating potential for controlled drug delivery and theranostic applications. Moreover, a composite ZIF-8 system (BNZ@ZIF-8; BNZ, Trypanosoma cruzi, and Benznidazole) was developed for controlled drug delivery, testing its release under various pH conditions. In vitro cell viability and cytotoxicity assays showed no significant toxic effects, and dissolution studies indicated the system could improve BNZ bioavailability through pH-sensitive release [25]. ZIF-8, a zinc-based MOF, has been widely studied for pH-responsive drug delivery, where it remains stable under physiological conditions but degrades in acidic tumor microenvironments to release encapsulated drugs such as doxorubicin. Such responsiveness enhances therapeutic efficacy while minimizing off-target effects, making MOFs promising candidates for precision medicine.

### 2.2. Biosensing and Molecular Recognition

**Figure 1:**
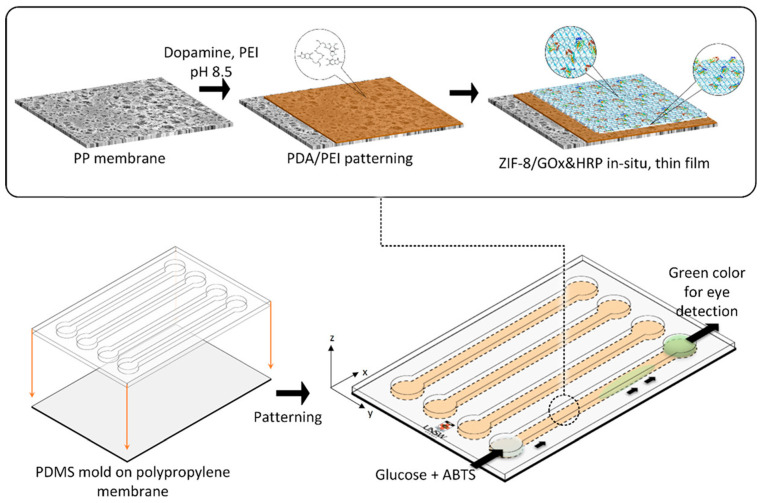
Illustration of the biocomposite patterning process. A bare polypropylene (PP) membrane is first patterned with a PDA/PEI coating using a PDMS mold containing microchannels. Subsequently, ZIF-8 is co-precipitated with the enzymes glucose oxidase (GOx) and horseradish peroxidase (HRP) directly onto the PDA/PEI layer, enabling in situ growth of the ZIF-8/enzyme composite. Copyright 2019 ACS [27].

In biosensing applications, MOF can be engineered to selectively capture and interact with a wide range of biological targets, including proteins, nucleic acids, and small molecules. When integrated into microfluidic devices, these frameworks enable rapid, sensitive, and multiplexed detection within a miniaturized and automated environment, significantly reducing sample and reagent consumption [13]. Recent advancements have demonstrated the utility of MOF-based microfluidic chips in detecting disease biomarkers, monitoring enzymatic activity, and enabling real-time analysis of cellular secretions [26]. Mohammad et al. developed enzyme-MOF composites via biomineralization to enhance enzyme stability while preserving catalytic activity [27]. Building on this concept, a patterning technique was employed to immobilize ZIF-8-based enzyme composites glucose oxidase (GOx) and horseradish peroxidase (HRP) onto flexible substrates within microfluidic channels using a polydopamine/polyethyleneimine (PDA/PEI) coating (Figure 1). The resulting glucose biosensor exhibited high stability, excellent selectivity, a broad linear range (8 μM–5 mM), and a low detection limit of 8 μM. This approach facilitates robust integration of enzyme–MOF materials into microfluidic biosensors and holds promise for broader biomolecular immobilization.

Complementing this strategy, Karimian et al. introduced a multifunctional system integrating a bio-MOF-based enantioselective recognition probe with a PDMS centrifugal microfluidic chip and a portable, smartphone-assisted colorimetric sensor for detecting L-tryptophan (L-Trp) [28]. By engineering oval-shaped gold nanoparticles with controlled morphology and surface passivation, the system achieved high chiral selectivity. Designed for user-friendliness and minimal reagent consumption, the device enabled fast, real-time measurement of L-Trp over a broad linear range (from 50 μM to 1.5 mM) with a detection threshold as low as 15 μM, even in complex matrices like serum, plasma, milk, and urine. These findings underscore the adaptability and promise of MOF-integrated microfluidic systems in biosensing applications across clinical, environmental, and food safety domains.

**Figure 2:**
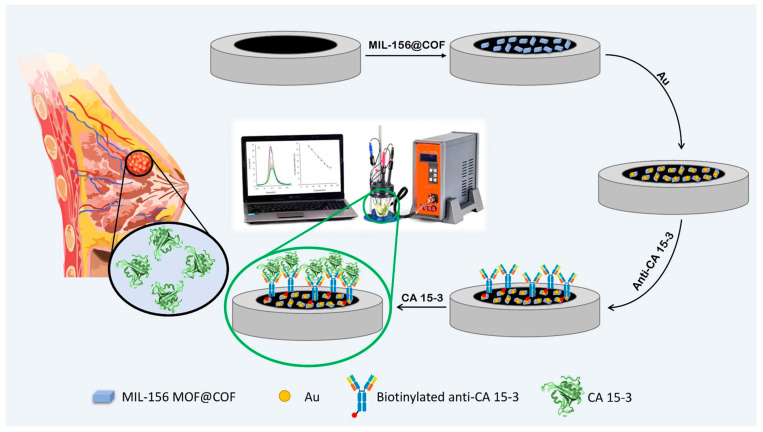
Schematically presentation of developed immunosensor. Copyright 2024 *Scientific Reports* [29].

Moreover, integrating MOFs with nanomaterials and signal amplification techniques has further improved detection limits and response times, paving the way for portable, point-of-care diagnostic systems with clinical relevance. Dezhakam et al. designed an ultrasensitive electrochemical biosensor for detecting CA15-3; the fabrication was carried out by combining MIL-156 MOF@COF nanocomposite with gold (Au) nanoparticles [29]. The biosensor was fabricated by modifying a glassy carbon electrode with MIL-156 MOF@COF and Au nanoparticles (Figure 2), enhancing the surface area, conductivity, and antibody binding. Using DPV, the device exhibited a linear detection range of 30–100 nU/mL for CA15-3 and successfully differentiated breast cancer patients from healthy individuals in serum, demonstrating its strong potential for clinical diagnostics.

Building on these advancements in MOF-based biosensors for protein markers like CA15-3, similar strategies are being extended to the detection of nucleic acid biomarkers such as RNA tumor markers, where signal amplification plays a critical role in overcoming detection challenges due to their low abundance [30]. RNA tumor markers have emerged as promising tools for cancer diagnosis and prognosis due to their association with abnormal gene expression. However, their low abundance poses a significant detection challenge [31]. Enhancing signal amplification is crucial in biosensor design to address this issue, particularly for sensitive and selective detection. Recent advances in electrochemical, photoelectrochemical, and fluorescent biosensors have enabled the precise identification of these biomarkers through innovative signal amplification strategies [32]. These biosensing platforms hold great potential for early cancer diagnosis and monitoring, which could lead to improved patient outcomes and reduced mortality rates.

#### 2.2.1. Signal Amplification

**Figure 3:**
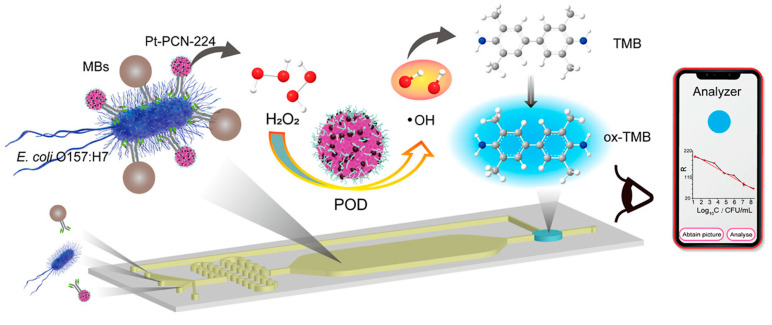
Schematic representation of microfluidic biosensing for foodborne bacteria using Pt-PCN-224 as a peroxidase-like catalyst for signal amplification. Copyright 2023 *ACS* [33].

Rapid identification of foodborne pathogens is essential for maintaining food safety and preventing public health crises. In a study by Xing et al., a zirconium-based metal–organic framework (Zr-MOF), enhanced with platinum nanoparticles (Pt-PCN-224), was developed to function as a peroxidase-mimicking signal enhancer for use in microfluidic biosensors (Figure 3) [33]. Using *Escherichia coli* O157:H7 as the target, the sensor demonstrated a broad linear detection range from 2.93 × 10^2^ to 2.93 × 10^8^ CFU/mL and achieved a remarkably low detection limit of 2 CFU/mL. The complete assay was carried out within a fully integrated microfluidic chip, allowing for fast and streamlined pathogen analysis. The system was tested on actual samples—including water, milk, and cabbage—and showed strong agreement with conventional culture techniques, achieving recovery rates between 90% and 110%. This work highlights the promise of MOF-based nanozyme platforms for delivering rapid, accurate, and on-site detection of bacterial contaminants in food safety monitoring.

Similarly, recently a portable microfluidic device was developed for rapid point-of-care detection of circulating tumor cells (CTCs) in pheochromocytoma (PCC) [34]. Using Au@CuMOF nanozymes and Fe_3_O_4_@SiO_2_ probes for dual recognition, the system enables glucose meter-based and colorimetric detection via enzyme-mimicking activity. Integrated with a smartphone app and glucose meter, it detects CTCs in whole blood with a 3 cells/mL limit and supports six-sample analysis, offering a low-cost, high-throughput alternative to traditional diagnostics.

#### 2.2.2. On-Chip Bacterial/Viral Detection

Detecting pathogenic bacteria is essential for safeguarding public health and ensuring the safety of food and water. Aptamer@MOF biosensors combine the target specificity of aptamers with the tunable porosity and stability of metal–organic frameworks (MOFs), enabling sensitive, selective, and real-time detection [35]. Building on this concept, Lu et al. developed the MAEA biosensor, which integrates magnetic Fe_3_O_4_@MOF@aptamers with an entropy-driven fluorescence system and mesophilic Clostridium butyricum Argonaute (CbAgo) enzyme [36]. In this system, phosphorylated aptamers anchored on zirconium-based MOF nanoparticles selectively capture bacterial targets and initiate an entropy-driven DNA circuit to generate guide DNA. CbAgo, directed by these guides, performs precise endonuclease activity, allowing rapid, amplification- and extraction-free detection of multiple pathogens with high sensitivity (<10^2^ CFU/mL). This approach presents a powerful, selective, and user-friendly platform for multiplexed bacterial diagnostics.

Complementing this, a label-free electrochemical genosensor was recently developed using a Zn-MOF/carboxymethyl cellulose (CMC) composite and Au nanoparticles deposited on a gold electrode to detect *Haemophilus influenzae* DNA in human plasma [37]. This system achieved an impressive detection limit of 1.48 fM and a wide linear range (0.1 pM–10 nM), with excellent specificity against mismatched and non-target sequences. The sensor demonstrated reliable performance and high recovery rates (98.4–103%), confirming its potential for accurate nucleic acid detection in clinical settings.

In addition to diagnostics, MOF-based materials are playing a transformative role in therapeutic applications, particularly in addressing challenges such as chronic wound infections [38]. A notable example is a multifunctional hydrogel dressing developed from a curcumin-based MOF (QCSMOF-Van), which is loaded with vancomycin and coated with quaternary ammonium chitosan [39]. This hydrogel effectively captures and eradicates bacteria through electrostatic interactions, aided by the synergistic antibacterial effects of Zn^2+^ ions and vancomycin. Beyond its antimicrobial activity, dressing also promotes tissue regeneration by modulating macrophage polarization and stimulating nerve and blood vessel growth. This dual-action system offers a comprehensive approach for infection management and accelerated wound healing, underscoring the versatile therapeutic potential of MOF-based platforms in modern biomedicine.

MOF-based microfluidic chips offer significant advantages in biomedical applications, including diagnostics, organ-on-a-chip systems, and point-of-care testing (POCT) [40]. In diagnostics, MOF-integrated chips facilitate rapid detection of biomarkers, pathogens, and small molecules, offering real-time analysis with minimal sample volume. Ilacas et al. reported two microfluidic paper-based analytical devices a well-based format and a lateral flow assay for glucose detection via a colorimetric assay [41]. Utilizing the MOF Zr-PCN-222(Fe) to encapsulate glucose oxidase, both platforms enable a visible brown color change upon reaction with glucose and KI, with signal intensity correlating to glucose concentration. These results demonstrate the effectiveness of MOF-based enzyme immobilization for advancing low-cost, portable biosensing technologies.

Furthermore, for POCT, the combination of MOFs with microfluidics enables portable, cost-effective, and user-friendly devices that can deliver accurate results outside traditional laboratory settings [42]. These innovations collectively demonstrate the transformative potential of MOF-based microfluidic chips in advancing personalized and accessible health care. In addition to diagnostic applications, MOF-based microfluidic platforms are increasingly utilized in organ-on-a-chip systems, enhancing physiological relevance for advanced drug screening and disease modeling.

### 2.3. Bioimaging Enhancement

MOFs can be engineered to incorporate luminescent metal ions or fluorophores, enabling their use in microfluidic chips for real-time, high-resolution imaging with precise spatial and temporal control [43]. For instance, lanthanide integrated MOFs exhibit strong luminescence, prolonged emission lifetimes, and low background noise, making them ideal for visualizing cellular and molecular processes [44]. Their modular architecture also supports post-synthetic functionalization with targeting ligands, enhancing selectivity in applications like cancer imaging [45]. Integrating MOFs into microfluidic systems improves imaging efficiency and consistency, while also reducing reagent usage [46]. Foucault-Collet et al. developed novel near-infrared (NIR)-emitting nano-MOFs incorporating Yb^3+^ ions and phenylene-based sensitizers, enabling their use in live-cell imaging [47]. Specifically, we introduced nano-Yb-PVDC-3, a structurally characterized and biologically stable MOF that resists photobleaching and exhibits an IC_50_ of 100 μg/mL. Confocal microscopy and ICP analysis confirmed its cellular uptake and cytoplasmic localization. Despite low quantum yield, its NIR emission was strong enough for imaging HeLa and NIH 3T3 cells, with clear distinction from cellular autofluorescence. This demonstrates the potential of Yb^3+^-based nano-MOFs for NIR biological imaging with single-photon excitation.

**Figure 4:**
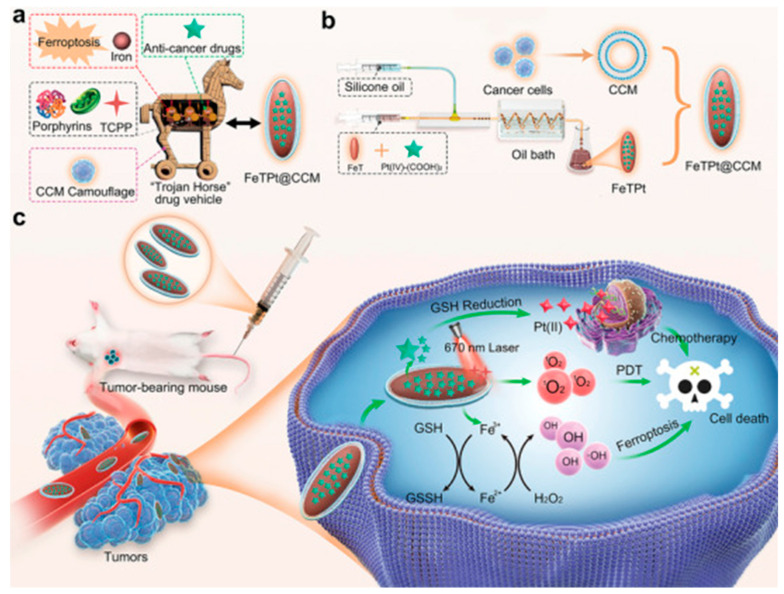
Schematic of FeTPt@CCM synthesis and its combined therapeutic functions. (**a**) Design of the bioinspired “Trojan Horse” nanocarrier. (**b**) FeTPt prepared via microfluidics and coated with cancer cell membrane (CCM). (**c**) After injection, FeTPt@CCM accumulates in tumors, where Pt(IV) is reduced to Pt(II) for chemotherapy, Fe^3+^ induces ferroptosis via GSH/H_2_O_2_ reactions, and TCPP generates ^1^O_2_ under 670 nm light for PDT. This system enables synergistic chemo-, ferroptosis, and photodynamic therapy. Copyright 2023 *Advanced Science* [48].

Building on the biomedical potential of MOFs demonstrated in imaging applications, recent advances have also explored their role as multifunctional therapeutic platforms for cancer treatment. Zhang et al. reported the development of MOF-based drug delivery systems show promise in cancer therapy, especially for multimodal treatments (Figure 4) [48]. Here, a biomimetic MOF nanocarrier (FeTPt@CCM) is developed via microfluidics, combining Fe^3+^, porphyrin, and an oxaliplatin prodrug, and coated with a cancer cell membrane for targeted delivery. Once inside tumor cells, it triggers ferroptosis and enhances photodynamic therapy through redox and Fenton-like reactions. Together with chemotherapy, FeTPt@CCM exhibits strong synergistic effects, effectively inhibiting cancer cell growth and tumor progression, highlighting its potential for advanced cancer treatment. Consequently, MOF-based microfluidic platforms offer promising potential for non-invasive, sensitive, and multiplexed bioimaging in both biomedical research and clinical settings.

**Figure 5:**
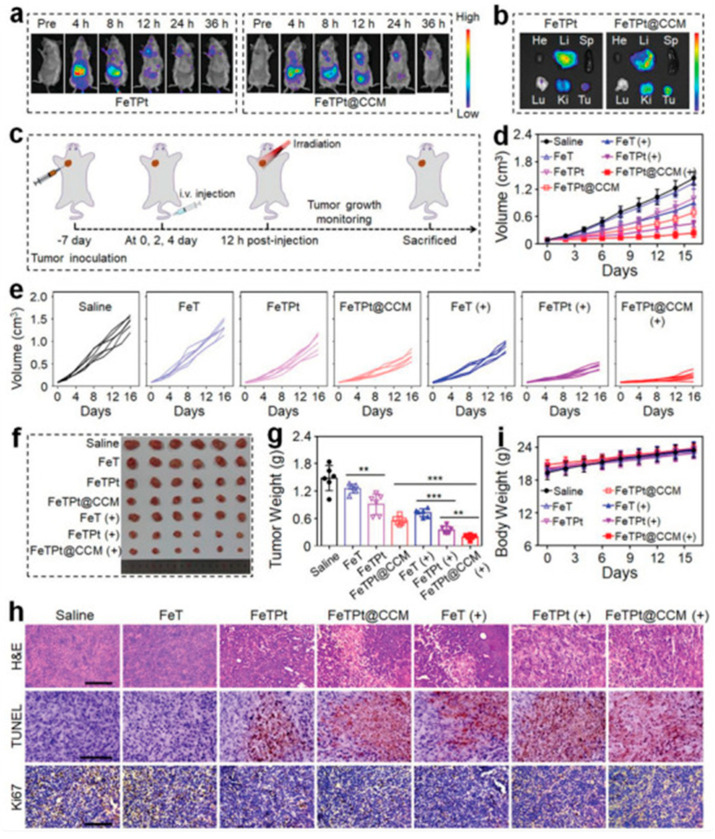
Evaluation of the in vivo anticancer efficacy of FeTPt@CCM. (**a**,**b**) Fluorescence imaging of tumor-bearing mice and excised organs/tumors following intravenous injection of FeTPt or FeTPt@CCM. (**c**) Overview of treatment schedule. (**d**,**e**) Tumor growth curves under various treatments. (**f**,**g**) Photographs and weights of tumors collected at study endpoint. (**h**) H&E, TUNEL, and Ki67 staining of tumor tissues (scale bar = 100 µm). (**i**) Body weight monitoring during treatment. Groups with (+) or without (−) irradiation; *n* = 6 per group. Data shown as mean ± SD; ** *p* < 0.01, *** *p* < 0.001 (“***” represents statistical significance). Copyright 2023 *Advanced Science* [48].

The potent in vitro anticancer activity of FeTPt@CCM led to in vivo evaluation of its biodistribution in breast tumor-bearing mice using ICG-labeled nanoparticles and IVIS imaging. As shown in Figure 5, both FeTPt and FeTPt@CCM accumulated at tumor sites, peaking at 12 h post-injection, with FeTPt@CCM exhibiting significantly stronger fluorescence. This enhanced tumor targeting is attributed to the cancer cell membrane (CCM) coating, which enabled homotypic recognition. Biodistribution analysis confirmed higher accumulation of FeTPt@CCM in tumors, liver, and kidneys, with intratumoral platinum content 1.53 times greater than FeTPt at 12 h, demonstrating its superior tumor-targeting efficiency.

**Figure 6:**
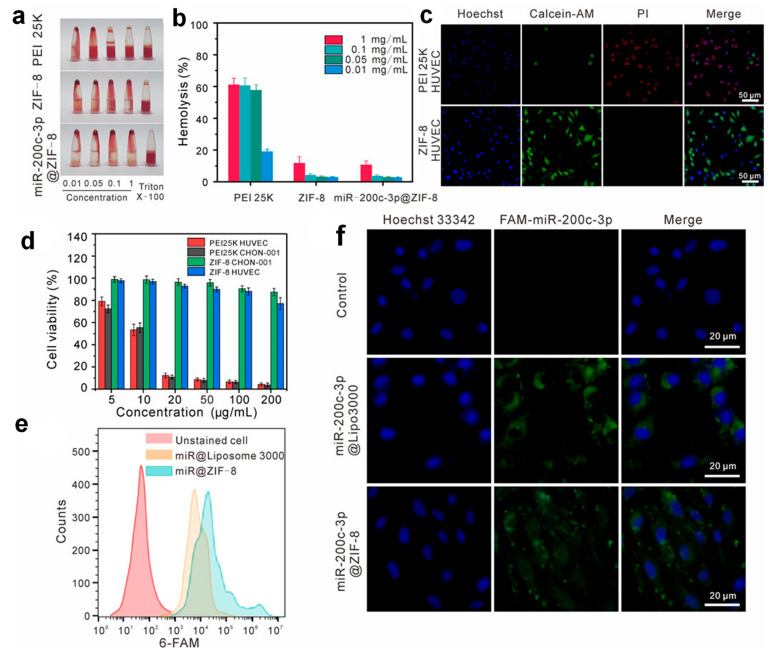
Assessment of cytotoxicity and uptake of miR-200c-3p@ZIF-8. (**a**,**b**) Hemolysis comparison of PEI 25K, ZIF-8, and miR-200c-3p@ZIF-8. (**c**) Live/dead staining of HUVEC cells after treatment. (**d**) Cell viability across concentrations of PEI 25K and ZIF-8. (**e**) Uptake analysis via flow cytometry. (**f**) Confocal imaging of CHON-001 cells treated with free and formulated miR-200c-3p. Copyright 2023 *Frontiers* [51].

Expanding the therapeutic potential of MOF-based nanoplatforms, recent research has turned toward gene regulation approaches, such as miRNA delivery, to tackle diseases beyond cancer [49]. MicroRNAs (miRNAs) are promising gene regulators for disease therapy but face challenges like toxicity, low uptake, and endosomal entrapment [50]. To overcome these, recently biocompatible ZIF-8 was used to deliver miR-200c-3p via a one-step Y-shaped microfluidic synthesis, yielding miR-200c-3p@ZIF-8 with high encapsulation efficiency, uniform size, and low toxicity [51]. ZIF-8 and miR-200c-3p@ZIF-8 showed low hemolysis (<20% at 1 mg/mL) and high biocompatibility, with no cytotoxicity and enhanced proliferation in CHON-001 and HUVEC cells, confirming their safe use for miRNA delivery (Figure 6a–d). The cellular uptake and transfection efficiency of miR-200c-3p@ZIF-8 were evaluated using flow cytometry and RT-qPCR. Both miR-200c-3p@ZIF-8 and Lipo3000 showed effective uptake in CHON-001 cells, with strong intracellular fluorescence (Figure 6e,f) and significantly elevated miR-200c-3p expression, confirming efficient transfection. This platform demonstrated therapeutic efficacy in osteoarthritis, offering a scalable strategy for safe and efficient miRNA delivery.

### 2.4. Antibacterial and Antiviral Activity

MOF-integrated microfluidic chips have shown promising potential in developing antibacterial and antiviral surfaces, owing to their distinctive structural features and chemical versatility [52]. The high surface area, tunable porosity, and functionalizable frameworks of MOFs allow for the controlled release of antimicrobial agents such as metal ions (e.g., Ag^+^, Cu^2+^, Zn^2+^) or reactive oxygen species (ROS), which can effectively disrupt bacterial membranes and viral envelopes [53]. When embedded within microfluidic devices, MOFs provide a localized and sustained antimicrobial environment, crucial for point-of-care diagnostics, implantable devices, and biosensors [54]. Recent advancements have focused on MOF-based coatings that inhibit microbial adhesion and biofilm formation, thereby enhancing the sterility and longevity of biomedical devices. Surface colonization by microbes and subsequent biofilm development are responsible for approximately 75% of human infections and are often resistant to conventional antibiotic treatments [55]. Antibacterial coatings have thus emerged as a promising strategy to prevent biofilm formation without compromising the properties of the underlying material. In this context, Arenas-Vivo et al. developed a novel silver-based MOF (AgBDC) with strong antifouling properties capable of inhibiting both bacterial adhesion and surface contamination [56]. The stability and biocidal efficacy of AgBDC against E. coli were confirmed through agar diffusion and colony counting assays. When applied as a spin-coated thin film, AgBDC effectively suppressed biofilm formation, as demonstrated by optical density measurements, colony counts, and confocal microscopy using calcofluor staining.

On the concept of multifunctional antibacterial surfaces, recent studies have also explored the integration of self-healing capabilities into MOF-based coatings to further extend their durability and effectiveness in harsh environments. A self-healing antifouling coating was developed using an MZ-8/MXene nanocomposite in a polyurethane matrix [57]. Triggered by NIR light, it achieved over 92% repair efficiency and reduced bacterial and algal adhesion by over 99% and 93%, respectively. The coating also offered strong anti-corrosion protection, making it suitable for marine and biomedical use.

**Figure 7:**
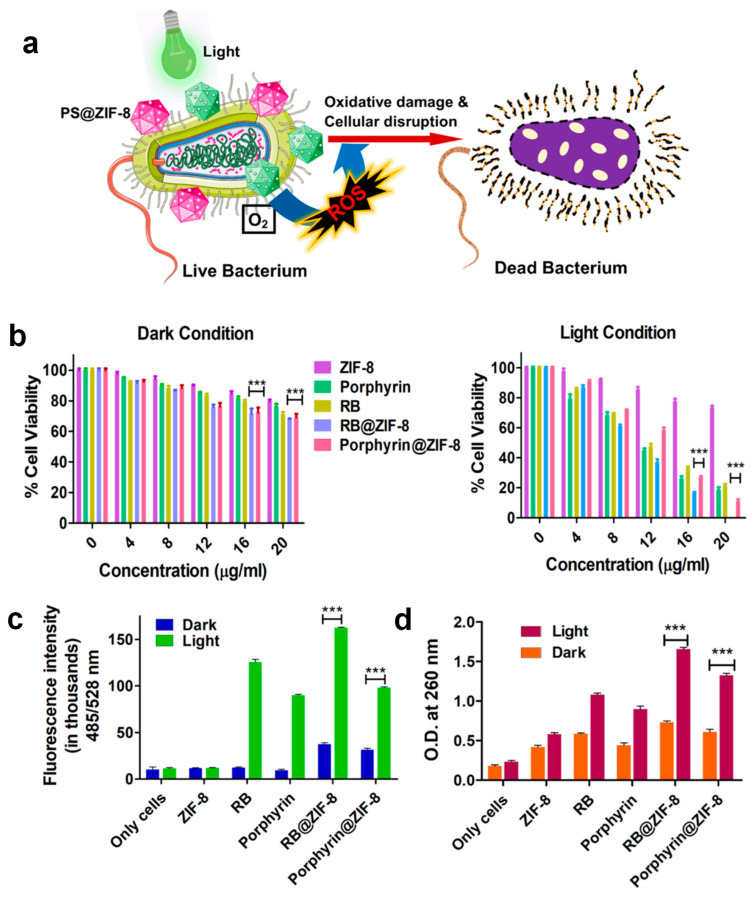
Antimicrobial activity of PS@ZIF-8. (**a**) Schematic showing microbial disruption by PS@ZIF-8. (**b**) Viability of *E. coli* after treatment with RB, porphyrin, ZIF-8, RB@ZIF-8, and porphyrin@ZIF-8 under dark and light conditions. (**c**) ROS generation assessed via DCFDA in treated and untreated cells under both lighting conditions. (**d**) Nucleic acid leakage from *E. coli* following probe exposure under dark and LED light (*** *p* < 0.001). Copyright 2025 ACS [58].

Moreover, the integration of photoresponsive and pH-responsive metal–organic frameworks (MOFs) enables stimuli-triggered antimicrobial action, making them highly adaptable to dynamic biological environments. To explore this potential, De et al. loaded two photosensitizers, Rose Bengal (RB) and porphyrin, onto Zeolite Imidazolate Framework 8 (ZIF-8) MOFs, achieving uniform particle sizes (~150 ± 50 nm) [58]. The RB@ZIF-8 complex generated more singlet oxygen and showed better pH-responsive behavior than other formulations. Additionally, RB@ZIF-8 exhibited a lower IC_50_ against *E. coli*, demonstrating its potential to combat multidrug-resistant bacteria. A conjectured mechanism illustrating the process is shown in Figure 7a, with the survival percentage (% survival) of *E. coli* for different probes depicted in Figure 7b, while exposure to green LED light revealed fluorescence intensity for RB, porphyrin, RB@ZIF-8, and porphyrin@ZIF-8, confirming the bactericidal effect through ROS formation (Figure 7c). Nucleic acid leakage studies, shown in Figure 7d, were conducted alongside ROS production to establish the antimicrobial photodynamic potential of the probes. This innovative approach addresses the challenges posed by drug-resistant pathogens, which are a growing threat to health care, and offers a solution to the limitations of antimicrobial photodynamic therapy (aPDT), including photobleaching and aggregation. These features collectively make MOF-based microfluidic surfaces a frontier in combating healthcare-associated infections and improving device biocompatibility.

#### Pathogen Detection and Antimicrobial Surfaces

Rapid pathogen detection and the development of antimicrobial surfaces are essential in controlling infectious diseases and preventing contamination [59]. Recent advancements in biosensors, such as microfluidic systems and MOFs, have enhanced detection sensitivity and speed. Meanwhile, antimicrobial surfaces, designed with antimicrobial agents or properties, offer promising solutions to reduce pathogen spread in health care and food safety [60,61]. This section highlights the latest innovations in detection methods and antimicrobial surface technologies [62]. Qi et al. reported a microfluidic biosensor using NH_2_-MIL-101(Fe) MOF with peroxidase-like activity was developed for rapid, sensitive *Salmonella* detection [63]. The system captures bacteria with immunomagnetic nanobeads, labels them with MOFs, and catalyzes a color change for quantification via a Raspberry Pi app 4 B (self-developed in Python). It detected *Salmonella Typhimurium* from 1.5 × 10^1^ to 1.5 × 10^7^ CFU/mL within 1 h, with a 14 CFU/mL detection limit and 112% recovery in spiked chicken samples. Its compact and automated design enables fast, reagent-efficient, and portable screening of foodborne pathogens.

Beyond detection, the ability to discriminate between live and dead bacteria is essential for clinical diagnostics, antimicrobial resistance monitoring, and informed therapeutic decisions [64]. MOF-based biosensors provide a promising solution for bacterial viability assessment due to their high surface area, tunable porosity, and ease of functionalization with selective biorecognition elements. For instance, a fluorescent biosensor constructed from Eu^3+^-doped MIL-53(Al) MOF and bacteriophage ligands enabled rapid detection of Pseudomonas aeruginosa within 15 min and effectively distinguished viable from non-viable cells in complex clinical samples [65]. This approach exemplifies the integration of MOF chemistry and phage-based specificity for advanced diagnostic applications.

Complementary strategies employing enzymatic activity have also emerged. Wu et al. reported a digital β-D-glucuronidase (GUS) assay embedded in a self-priming PDMS microfluidic chip for single-cell detection of viable Escherichia coli [66]. This platform discretizes individual bacteria into picoliter chambers, where the enzymatic activity of GUS serves as a proxy for cell viability. The method allows detection and quantification within 3–4 h, without requiring external instrumentation, and offers practical utility for evaluating antimicrobial performance, environmental safety, and infectious disease diagnostics.

Further expanding the scope, a digital GUS-AST assay using a 3D microwell array chip has been developed for direct viability detection and antimicrobial susceptibility testing (AST) of *E. coli* from urine samples [67]. By quantifying single-cell metabolic activity, the assay enables simultaneous estimation of bacterial load and determination of minimum inhibitory concentrations (MICs) within 4.5 h, offering a rapid, culture-free alternative to conventional AST protocols.

Similarly, another microfluidic 3D microwell platform was engineered to streamline single-cell AST directly from raw urine samples [68]. Featuring a capillary valve-based flow distributor and pre-loaded reagents, the device enables uniform sample partitioning and automated viability assessment without pre-culture steps. It delivers quantifiable MIC results in under 3 h, supporting faster, targeted antibiotic prescriptions and contributing to efforts in curbing antibiotic resistance.

Finally, rapid AST tools are urgently needed for life-threatening infections such as sepsis [69]. A recent high-throughput microfluidic platform employing [70]. MnO_2_@ZIF-90 nanoprobes exhibiting both nanozyme and fluorescent properties demonstrated dual-mode detection (colorimetric and fluorescent) with a detection limit of 10 CFU/mL and a turnaround time of less than 5 min. This approach underscores the clinical potential of integrating MOF-based nanozymes with microfluidic platforms for scalable, point-of-care AST.

## 3. Microfluidic Platforms in Biomedicine

### 3.1. Diagnostic and Therapeutic Platforms

The integration of MOFs into microfluidic systems has significantly advanced the development of next-generation diagnostic platforms [71]. These platforms capitalize on the precise fluid manipulation capabilities of microfluidics and the selective recognition or catalytic features of MOFs to enable rapid, low-volume, and high-throughput analyses [72]. MOF-based microfluidic chips have been successfully employed in the detection of nucleic acids, proteins, and pathogenic microorganisms, using fluorescence, electrochemical, or colorimetric readouts.

For instance, Chen et al. developed a 2D MOF-based Nano-DMFC platform that uses FRET nanosensors to detect dual miRNAs simultaneously in single breast cancer cells, accurately identifying 10 circulating tumor cells (CTCs) among 10,000 cells with high reproducibility in spiked blood samples [73]. Such platforms offer key advantages for point-of-care diagnostics, including portability, reduced assay times, and multiplexing capabilities. Consequently, MOF-integrated microfluidic diagnostic systems present significant potential for advancing clinical diagnostics, especially in decentralized and resource-limited healthcare environments.

#### 3.1.1. Organ-on-a-Chip

As the demand for effective preclinical drug testing increases, in vitro models such as organ-on-a-chip (OOC) systems have become essential tools for drug screening and disease modeling. In this context, metal–organic frameworks (MOFs) have emerged as valuable components in OOC platforms, capable of mimicking the extracellular matrix or enabling the controlled release of biochemical cues, thereby enhancing the replication of complex physiological microenvironments. For example, Kim et al. demonstrated the potential of MOF-integrated OOC systems for improved biomimicry [74]. Despite these advancements, bone-on-a-chip (BOC) models that accurately replicate the bone microenvironment remain underdeveloped. Considering the dynamic nature of bone tissue and the global rise in age-related bone disorders, BOC platforms hold significant promise for studying orthopedic diseases and evaluating drug responses without relying on animal models.

**Figure 8:**
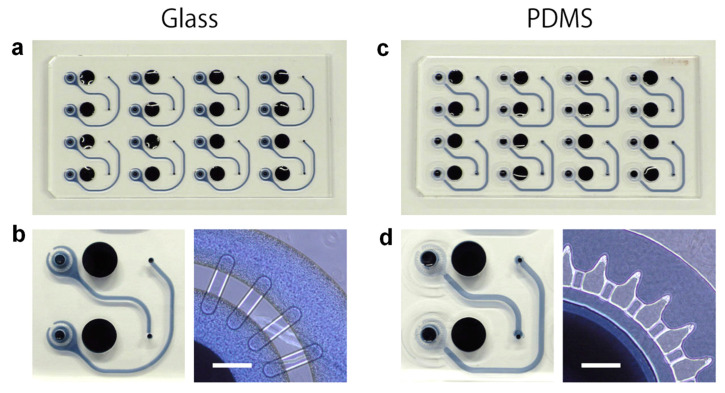
Organ-on-a-chip devices constructed from glass (**a**,**b**) and PDMS (**c**,**d**). (**a**,**c**) Full views of each device. (**b**,**d**) Close-up of a single culture chamber and Laplace valves. Scale bars: 500 μm. Copyright 2019 *Elsevier* [75].

To support the advancement of such platforms, material choice in OOC fabrication is critical. While polydimethylsiloxane (PDMS) is commonly used due to its ease of prototyping, it tends to absorb small hydrophobic molecules, leading to compound loss and reduced assay reliability. Addressing this limitation, Hirama et al. reported a glass-based OOC device was developed and evaluated against PDMS-based systems [75]. The glass device demonstrated stable medium flow, minimized molecular absorption, and enhanced cell adhesion, offering a more robust and accurate platform for assessing small hydrophobic compounds in cell-based assays. Figure 8a,b illustrates the fabrication of glass and PDMS devices. Although the glass design was slightly modified due to fabrication constraints, the final structure remained a stable microfluidic system (Figure 8c,d).

#### 3.1.2. Key Performance Metrics

Key performance parameters such as flow control, detection sensitivity, and miniaturization are pivotal for the effective application of MOF-based microfluidic chips in biomedical contexts [76,77]. Flow control ensures precise manipulation of fluids within microchannels, a critical aspect for accurate diagnostics and reliable drug testing [78]. Advances in integrated microfluidic components, including valves and pumps, have significantly improved fluid dynamics control, enhancing system performance [79]. Detection sensitivity is another crucial factor, as it dictates the platform’s ability to detect low-abundance biomarkers or pathogens [80]. Ling et al. developed a nano-metalloporphyrinic MOF (NporMOF(Fe)) with high surface area, exposed Fe sites, and strong catalytic activity was developed as an artificial enzyme for nonenzymatic electrochemical sensing [81]. The NporMOF(Fe)-modified electrode enabled sensitive detection of NO (5–200 μM, LOD: 1.3 μM) and H_2_O_2_, including real-time monitoring from HeLa cells. It also demonstrated high selectivity against biological interferents, highlighting its potential for advanced MOF-based biosensing applications. Additionally, miniaturization is essential for developing portable, cost-effective devices that are suitable for point-of-care applications [82]. By reducing device size while preserving or improving performance, miniaturization allows for broader use in clinical environments, facilitating rapid and efficient diagnostics [83]. Collectively, these performance parameters contribute to the ongoing advancement of MOF-based microfluidic platforms, enhancing their utility in biomedical applications.

### 3.2. Types of Microfluidic Chips

#### 3.2.1. Paper-Based

Microfluidic chip technologies have evolved into various formats based on the materials used, each offering distinct advantages for biomedical applications. Paper-based microfluidic chips are low-cost, biodegradable, and require no external power, making them ideal for point-of-care diagnostics in low-resource environments [84]. Recently, to address heavy metal contamination in food, a low-cost, self-driven paper-based microfluidic device (μPAD) was developed for the simultaneous detection of Pb^2+^, Hg^2+^, Cd^2+^, and As^3+^ [85]. Fabricated using wax and screen printing, the chip enabled pump-free fluid flow via capillary action. A fluorescence “turn-on” aptasensor and smartphone imaging allowed quantification through RGB analysis, with detection limits as low as 1.65–4.20 nM. Applied to apple and lettuce samples, the device achieved recovery rates of 84.0–104.1%, demonstrating its potential for real-time, on-site heavy metal monitoring.

Expanding beyond environmental monitoring, paper-based microfluidics are now being applied to biomedical diagnostics such as total antioxidant capacity (TAC) detection. Wu et al. developed a cost-effective POCT method that integrates a nanozyme-catalyzed colorimetric sensor with smartphone-based detection [86]. The nanozyme, synthesized through a solvothermal process, exhibited strong catalytic performance, with Michaelis–Menten constants of 0.11 mM for H_2_O_2_ and 0.129 mM for TMB. This system enables rapid TAC assessment in body fluids, including blood, saliva, and sweat, with a detection limit of 33.4 μM and a linear range of 50–700 μM, delivering results in under 15 min. The platform holds promises for portable biochemical analysis and POCT applications.

#### 3.2.2. Polymer-Based

**Figure 9:**
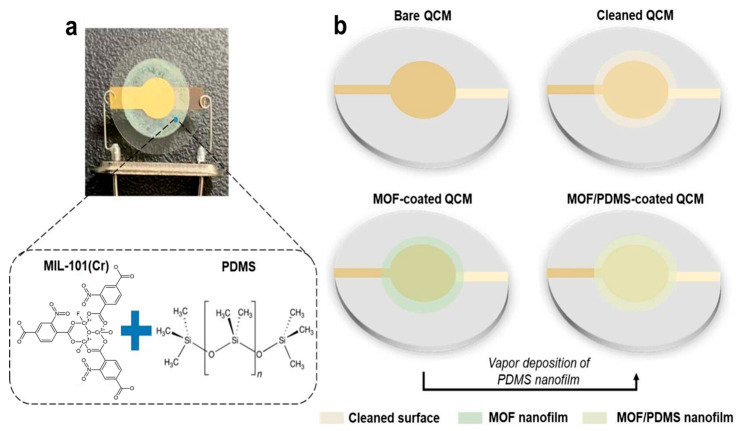
VOC sensors developed in this study. (**a**) Image of the QCM-based sensor coated with MOF/PDMS. (**b**) Schematic showing the fabrication process of the MOF/PDMS-coated sensor. Copyright 2024 *ACS* [87].

While paper-based microfluidics excel in low-cost POCT applications, other materials like Polydimethylsiloxane (PDMS) offer unique advantages for more complex biomedical and environmental sensing tasks. PDMS-based chips are the most widely used due to their biocompatibility, optical transparency, and ease of fabrication, allowing for rapid prototyping and real-time imaging of biological processes. Cao et al. developed a MOF-based quartz crystal microbalance (QCM) sensors offer promise for volatile organic compound (VOCs) detection but suffer in humid conditions due to MOFs’ hydrophilicity [87]. To address this, a virtual sensor array (VSA) using a MIL-101(Cr)/PDMS composite layer was developed, enhancing moisture resistance and maintaining stable performance after water exposure. The sensor achieved high sensitivity (2.68 mdB ppm^−1^) and low detection limits (20.06 ppm), accurately identifying four VOCs with 95.3% accuracy under 60–90% humidity, making it suitable for humid environments like underground spaces. Figure 9a shows the QCM VOC sensor featuring a PDMS-coated MOF film, while Figure 9b outlines its fabrication process.

Similarly, PMMA microfluidic chips have gained attention for separation applications. A simplified fabrication process using UV-initiated polymerization at room temperature avoids the need for complex equipment [88]. In this method, microchannel structures are imprinted from a silicon master using a sandwich mold, followed by thermal sealing. The approach produces high-quality, reproducible chips with smooth channel surfaces and enhanced electroosmotic flow (EOF). With the ability to generate nearly 100 chips from a single master, this method supports scalable manufacturing and compatibility with amperometric and contactless conductivity detection. Moreover, its applicability to other UV-curable materials makes it a practical and cost-effective strategy for producing advanced separation microchips. Together, these PDMS and PMMA-based approaches underscore the expanding role of alternative polymers in the development of high-performance MOF-integrated microfluidic platforms.

#### 3.2.3. Glass-Based Microfluidic Chips

Microfluidic chips made from glass are highly preferred in analytical fields because they provide excellent chemical durability, clear optical properties, and stable performance, which are essential for precise measurements. Nonetheless, their fabrication is often more complex and costly compared to polymer alternatives [89]. Among thermoplastics materials, polymethyl methacrylate (PMMA) and cyclic olefin copolymer (COC) are widely used due to their combination of mechanical strength and suitability for large-scale manufacturing processes, making them favorable for mass production of chips [90]. The choice of substrate material critically influences fluid dynamics, sensor integration, and strategies for immobilizing metal–organic frameworks (MOFs), thereby directly impacting the overall performance and applicability of MOF-based microfluidic systems in diagnostics, drug delivery, and biosensing [91].

In particular, glass-based biochips have attracted increasing attention over commonly used polymers like polydimethylsiloxane (PDMS) due to their enhanced chemical stability, biocompatibility, and optical clarity. Recent advances have demonstrated the feasibility of a glass molding process (GMP) applied to D-ZK3 glass for fabricating microstructures tailored to microfluidic devices [92]. This approach involved using molds with protruding features to evaluate GMP performance with respect to surface roughness and structural accuracy. Molds for representative chip designs—a diffusion mixer, a flow focusing chip, and a cell counting device—were fabricated and tested for wear resistance, confirming the durability of the molds. Functional validation was performed through a liquid-filling test of the molded chips. These findings establish that high-quality glass microstructures can be produced rapidly (within 12 min), underscoring GMP’s potential as a scalable fabrication method for microfluidic platforms.

Separately, wearable technologies such as Google Glass provide innovative opportunities for real-time biomedical monitoring. Google Glass is a head-mounted device that wirelessly displays information hands-free and supports remote control via voice commands, making it suitable for medical and biomedical applications [93]. An integrated system was developed combining hardware, firmware, software, and custom glassware to wirelessly transmit sensor data to Google Glass, enabling real-time visualization and bidirectional interaction. This platform was successfully applied to monitor temperature, pH, and morphology in liver- and heart-on-chip models, and to remotely control drug delivery within a microfluidic liver bioreactor. This advancement demonstrates the potential of wearable devices for enhancing monitoring and control capabilities in diverse biomedical systems.

## 4. Strategies for Integrating MOFs into Microfluidic Platforms

### 4.1. Fabrication Techniques

Integrating MOFs into microfluidic systems enhances biomedical devices by combining MOFs’ unique properties with the precision of microfluidics [94]. MOFs enable selective analyte capture, signal amplification, and controlled drug release. Integration methods include in situ growth, drop-casting, and surface immobilization [95]. These hybrid systems support sensitive biosensing, real-time diagnostics, and organ-on-a-chip models with low reagent use and high throughput [96]. Advances in MOF synthesis and microfabrication will further improve their stability, compatibility, and performance in next-generation biomedical devices [97].

#### 4.1.1. In Situ Growth

**Figure 10:**
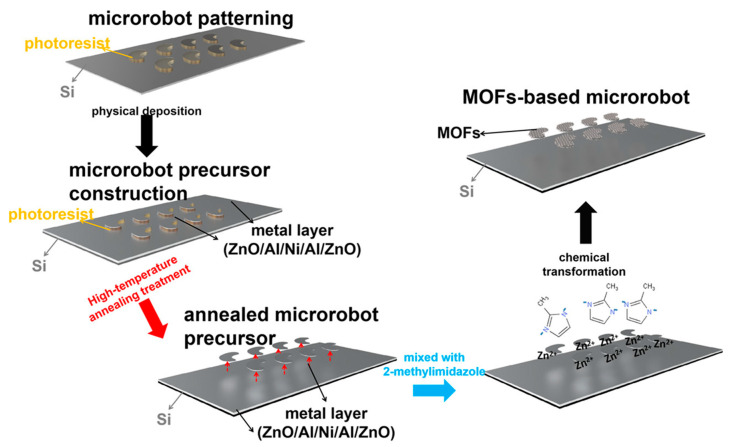
Diagram showing the process for creating MOF-integrated microrobots on silicon substrates. Copyright 2021 *ACS* [98].

Magnetic microrobots offer promising biomedical applications such as targeted therapy and minimally invasive procedures. Mu et al. this studied an optimized the crystallinity of ZnO on 2D microrobot precursors via annealing, enabling efficient growth of ZIF-8 MOF coatings (Figure 10). Annealing at ~410 °C enhanced surface roughness, promoting uniform MOF formation [98]. The resulting MOF-based microrobots demonstrated strong propulsion (up to 107 μm/s), precise navigation in microfluidic channels, and high drug loading capacity, highlighting their potential for targeted delivery in hard-to-reach areas of the body.

Moreover, circulating tumor cell (CTC) detection is limited by their low presence in blood and the inefficiency of current isolation methods. To address this, Kefayat et al. developed a CTC dialysis system combining (i) core–shell Cu–CuFe_2_O_4_@MIL-88A nanoparticles functionalized with anti-HER2 antibodies for targeted CTC capture, and (ii) a 3D-printed microfluidic filter with polycaprolactone/Fe_3_O_4_ composite under a magnetic field for efficient isolation from whole blood [99]. The system achieved over 80% capture efficiency, maintained CTC viability, and showed no adverse effects on normal blood cells, offering a promising tool for rapid, large-volume CTC processing.

#### 4.1.2. Layer-by-Layer (LBL) Assembly

Layers of MOFs and complementary materials are built up via self-assembly or electrostatic interactions [100]. Layer-by-layer (LBL) thin film assembly has evolved significantly through advancements in materials, fabrication techniques, and characterization methods. Emerging approaches, including hybrid systems, bio-integrated assemblies, and continuous “quasi”-LBL processes, are shaping the future of this field and expanding its potential for diverse applications [101]. In analytical chemistry, photonic sensors are emerging as powerful tools, especially in clinical diagnostics [102]. Among these, sensors based on photonic integrated circuits (PICs) are known for their high sensitivity [103]. When combined with microfluidics, these systems can analyze samples as small as a few microliters. However, their main limitation is low specificity. To address this, LBL assembly can be applied for precise surface modification, monitored through resonance shift measurements. Bovine serum albumin (BSA) and tannic acid (TA) were used to form stable LBL films via hydrogen bonding, with functional groups available for target conjugation. Silicon nitride-based micro-ring resonators (MRRs) and Mach–Zehnder interferometers (MZIs), integrated with microfluidics. This demonstrates the potential of PIC sensors for real-time surface modification control in point-of-care devices, aiding early disease detection and treatment monitoring.

To address the challenge of low specificity in photonic-based sensors, the application of LBL assembly provides a precise technique for surface modification [104]. This method can be seamlessly integrated with advanced fabrication processes, such as the fully automated LBL liquid-phase epitaxy (LPE) technique [105]. For example, Jiang et al. presented a study that emphasized the use of LBL-LPE for the controlled synthesis of two-dimensional MOFs. Utilizing nickel(II) and 2-aminoterephthalic acid (BDC-NH_2_) as precursors, uniform and crystalline Ni-MOF thin films were deposited on Si/SiO_2_ substrates within modular microfluidic chips [20]. The LBL-LPE approach enabled precise control over film thickness (2–25 nm) and ensured homogeneous growth. Integration onto metal microelectrode arrays (MEAs) demonstrated the scalability and reproducibility of the LBL method, positioning it as an effective strategy for fabricating MOF-based sensor arrays.

#### 4.1.3. Immobilization

MOFs are chemically bonded or physically adsorbed onto functionalized microfluidic surfaces [106]. Recent advancements in covalent enzyme immobilization within MOFs, covalent organic frameworks (COFs), and hydrogen-bonded organic frameworks (HOFs) have led to notable improvements in enzyme stability and activity [107]. This method improves enzyme loading efficiency but also significantly boosts catalytic performance and enables better enzyme re-usability, positioning MOFs, COFs, and HOFs as highly efficient platforms for enzyme immobilization.

DNA’s precise and programmable base pairing enables effective target recognition, while hydrogels provide a three-dimensional network that facilitates stable immobilization of biomolecules and isolates interfering substances [108]. This combination in DNA hydrogels enhances biosensor performance by improving target capture and signal reliability.

#### 4.1.4. Encapsulation in Polymers

MOFs are embedded in a biocompatible matrix that can be molded into microfluidic structures [109]. Rohra et al. reported a microfluidic strategy for fabricating glucose-responsive drug delivery systems by embedding insulin and gold nanoparticles (AuNPs) into ZIF-8 using a single-step continuous-flow synthesis [110]. Within the porous MOF, AuNPs exhibit glucose oxidase-like behavior by converting glucose into gluconic acid and hydrogen peroxide. The resulting pH drop triggers the degradation of ZIF-8, releasing the encapsulated insulin. This embedding approach ensures high loading efficiency (77% in batch, 88% in microfluidics) and preserves the structural and functional integrity of the MOF. The system highlights an efficient method for constructing stimuli-responsive, bioactive MOFs with potential in drug delivery and biosensing.

In addition to this, a biocompatible scaffold fabricated by embedding curcumin and Fe(II)-based MOFs into a polydimethylsiloxane (PDMS) matrix, forming a Cur/Fe-MOF/PDMS composite. The embedding strategy involved integrating the drug-loaded MOF within the porous PDMS sponge [111]. The scaffold exhibited a uniform porous structure ideal for drug delivery and tissue interfacing. In vivo results showed no adverse cytotoxic effects, with enhanced tissue regeneration and revascularization observed in the Cur/Fe-MOF/PDMS group, supported by H&E staining. A two-phase curcumin release profile was noted, with initial rapid release attributed to the hydrophilic feature of the MOF. These findings highlight the composite’s potential for applications in the microfluidic or biomedical field, including implantable therapeutic platforms and 3D tissue engineering.

Expanding on MOF-based scaffold innovations, microfluidic strategies have proven highly effective for the precise fabrication of functional MOF hollow spheres. These methods allow for the accurate encapsulation of bioactive agents, maintaining their functionality and boosting their effectiveness in biomedical uses. Jeong et al. reported a functional bio-MOF wherein hollow spheres ranging from 35 to 2000 μm in size were efficiently synthesized through a single-step, continuous-flow droplet microfluidic approach via interfacial reaction [112]. The MIL-88A framework was tailored into both single- and double-layered hollow configurations. Furthermore, a variety of functional nanoparticles, including silica, cobalt, and UiO-66(Zr) MOFs, were incorporated into the single-shell spheres without compromising their original properties. Importantly, three enzymes—glycerol dehydrogenase, horseradish peroxidase, and acetylcholinesterase—were successfully encapsulated under mild conditions, retaining their enzymatic functions and demonstrating improved reusability compared to unbound enzymes.

Additionally, thermally assisted microfluidic systems facilitate the scalable and uniform production of biocompatible MOF-based nanocomposites, enhancing their potential for diverse biomedical applications. A thermally assisted platform was developed to continuously fabricate drug-loaded MOFs@SiO_2_ nanoparticles, achieving uniform morphology, narrow size distribution, improved structural stability, and excellent biocompatibility [113]. This adaptable approach can encapsulate a variety of therapeutic agents and dyes. The successful use of Rose Bengal-loaded nanoparticles in vivo photodynamic cancer therapy highlights the significant promise of this strategy in biomedical fields.

#### 4.1.5. 3D Printing Approaches

MOFs are mixed with printable inks or resins for direct fabrication of device components. Chang et al. reported a disulfiram (DSF), a drug under clinical evaluation for cancer therapy, in combination with copper (Cu) ions to form the antitumor complex diethyldithiocarbamate copper [Cu(DDC)_2_] [114]. However, the separate formulation of DSF and Cu results in low tumor concentrations and reduced efficacy. To address this, we developed a novel biomimetic nanoparticle formulation of Cu(DDC)_2_ using the stabilized metal ion ligand complex (SMILE) method, creating metal–organic nanoparticles (MONs) with a Cu(DDC)_2_ core and BSA shell. A 3D-printed microfluidic platform was employed to enhance the fabrication process of MONs, ensuring accurate control and scalability. The resulting BSA/Cu(DDC)_2_ nanoparticles showed strong anticancer activity in both cell-based and animal studies, underscoring their potential to enhance DSF/Cu-based cancer treatments.

In parallel, a 3D printing technique was employed to construct a microfluidic electrochemical sensor with precise architectural control. The internal fluid dynamics of the device were optimized using the finite element method (FEM) [115]. A flexible screen-printed electrode (SPE), enhanced with porous Mn_2_O_3_ derived from a manganese-based MOF (Mn-MOF), was integrated into the system, greatly improving its electrochemical response. This microfluidic sensor demonstrated excellent sensitivity for detecting heavy metal ions, with linear calibration ranges of 0.5–8 μg/L for Cd(II) and 10–100 μg/L for Pb(II). The detection limits achieved were 0.5 μg/L for cadmium and 0.2 μg/L for lead, both significantly below the safety limits set by the World Health Organization. These findings underscore the effectiveness of the fabrication method and the superior physical properties of the MOF-derived electrode material for accurate and reliable heavy metal ion detection.

MOFs are loaded onto wax-printed or laminated chromatography paper for paper-based microfluidic devices (μPADs). The integration of MOFs with paper substrates has gained increasing attention for advancing POCT technologies, particularly in biomedical diagnostics. Paper-based platforms offer cost-effectiveness, portability, and ease of fabrication, making them ideal for resource-limited settings. Functionalizing these substrates with MOFs highly porous and tunable nanomaterials significantly enhances their sensitivity, selectivity, and multifunctionality. This MOF–paper synergy is increasingly being incorporated into microfluidic chip designs, enabling compact, efficient, and user-friendly diagnostic devices for biomedical applications such as disease biomarker detection, environmental toxin monitoring, and real-time health screening. Recent innovations demonstrate the potential of MOF-functionalized paper microfluidic systems to support rapid analysis with minimal sample volumes, offering promising prospects for decentralized health care and personalized medicine.

#### 4.1.6. Hybrid Systems

MOFs offer significant potential for biomedical applications, yet their cytotoxicity and biocompatibility must be rigorously assessed. This includes detailed investigations of cellular uptake, biodistribution, and long-term effects in both in vitro and in vivo settings [116]. To improve safety and inform the deliberate development of MOF-based microfluidic systems for medical applications, strategies such as surface modification, encapsulation, and computational modeling are critical [117]. Wagner et al. developed real-time sensor-based and cellular screening approaches; these approaches were employed to assess the toxicity of two widely used functionalized MOFs, MIL-160 (hydrophilic) and ZIF-8 (hydrophobic), which were synthesized in-house [118]. These materials were tested on human bronchial epithelial (BEAS-2B) cells, a model relevant for toxicology studies. The screening process allowed for the high-throughput analysis of cellular responses, highlighting differences in toxicity mechanisms and exposure pathways. The study underscores the necessity of thorough toxicological evaluations—particularly considering surface functionalization—before implementing MOFs in biomedical settings. The findings also provide important insight into how MOFs affect cell viability, morphology, and interactions with substrates.

### 4.2. Compatibility with Chip Materials and Fabrication Constraints

In the development of MOF-based microfluidic chips for biomedical applications, ensuring compatibility with chip materials and fabrication techniques is essential for optimizing device performance and long-term stability [119]. Common materials employed in microfluidic chip fabrication, such as polydimethylsiloxane (PDMS), glass, and silicon, must facilitate efficient integration with MOFs while maintaining the structural integrity and functionality of both components [120]. PDMS, widely used due to its flexibility and transparency, presents challenges due to its hydrophobic nature, which can impede effective MOF attachment or interaction with aqueous biological samples [121]. In contrast, glass and silicon provide enhanced chemical stability and superior surface properties for MOF deposition, though they often necessitate more intricate processing techniques. Various fabrication strategies, including direct deposition, layer-by-layer assembly, and photolithography, must be optimized to achieve uniform MOF incorporation while avoiding aggregation, which can compromise the chip’s functionality [122,123]. Moreover, surface modification approaches such as silanization or polymer functionalization are often employed to enhance the interaction between the MOFs and chip materials, ensuring better stability, higher sensitivity, and improved overall performance in biomedical applications [124,125].

## 5. Challenges and Future Directions

### 5.1. Limitations of MOF-Based Microfluidic Chips in Biomedical Applications

Despite the significant promise of MOF-based microfluidic chips for biomedical applications, their practical implementation remains constrained by a range of critical limitations [126]. One of the foremost challenges is ensuring the chemical and structural stability of MOFs under biologically relevant conditions, including aqueous environments, varying pH levels, and complex physiological media, which can lead to hydrolysis, surface degradation, or loss of structural integrity [127,128,129]. Biofouling caused by non-specific adsorption of proteins, cells, or microbes further compromises device performance, reducing sensitivity and reliability [130]. In addition, the biocompatibility and potential toxicity of MOFs must be thoroughly assessed, as the leaching of metal ions or organic linkers may pose safety risks in clinical settings [131].

The integration of MOFs into confined microscale channels presents further technical challenges, including achieving uniform distribution, stable adhesion, and long-term functionality [132]. Moreover, scalability and reproducibility of device fabrication remain major obstacles to large-scale production and consistent performance across batches [133]. Regulatory barriers add another layer of complexity, as comprehensive evaluation protocols for safety, stability, and efficacy must be established [134]. These limitations underscore the urgent need for continued interdisciplinary research aimed at developing robust, biocompatible MOFs, standardized integration methods, and validated regulatory frameworks to facilitate the clinical translation and commercial deployment of MOF-based biofluidic systems [135,136,137].

### 5.2. Future Directions and Opportunities

The integration of MOFs into microfluidic platforms has ushered in a new era of innovation in biomedical applications [138]. Rather than relying solely on static materials, current research focuses on responsive, multifunctional systems capable of real-time interaction with biological environments [139]. Emerging fabrication techniques, data-driven design, and an increasing push toward clinical translation are shaping the direction of the field [140]. In particular, advancements in smart materials, additive manufacturing, artificial intelligence, and regulatory alignment are collectively steering MOF-based microfluidics toward greater sophistication and real-world applicability [141].

#### 5.2.1. Smart and Stimuli-Responsive MOFs

Stimuli-responsive or “smart” MOFs have become central to the evolution of biomedical microfluidics [142]. These materials exhibit dynamic behavior in response to environmental triggers such as pH fluctuations, temperature changes, light exposure, redox states, and specific biomolecules. For example, pH-sensitive MOFs can be engineered to release drugs in acidic tumor microenvironments, while thermo-responsive MOFs support temperature-dependent drug delivery [143]. Photoresponsive MOFs further enable precise control through light-activated reactions, applicable in biosensing and phototherapy [144]. Their integration into microfluidic chips enhances device functionality by allowing selective and timely responses, thereby improving therapeutic precision and diagnostic sensitivity in point-of-care and personalized medicine contexts.

#### 5.2.2. AI-Assisted MOF Sensor Design

**Figure 11:**
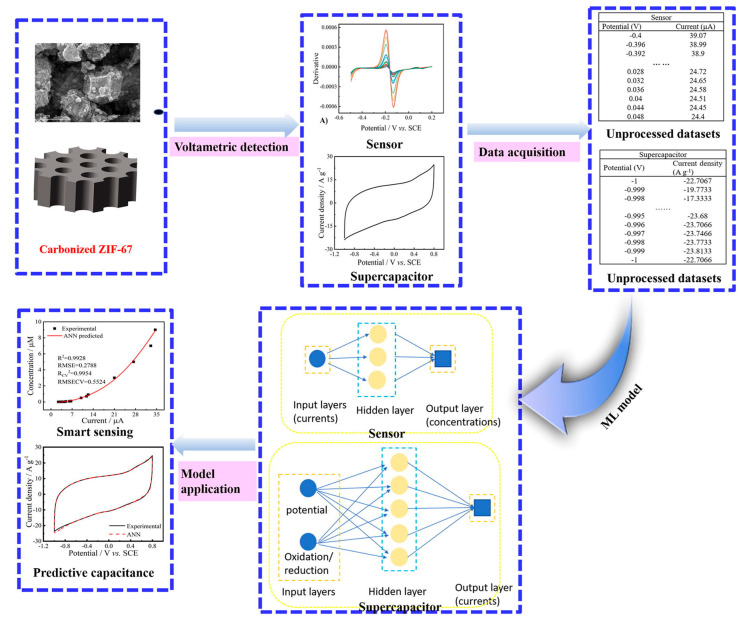
Schematic of the artificial neural network (ANN)-assisted design and implementation of a dual-function system for sensing and supercapacitor applications. Copyright 2022 *Elsevier* [148].

The incorporation of artificial intelligence (AI) into the development of MOF-based microfluidic biosensors is proving to be a transformative approach, significantly boosting their accuracy, efficiency, and performance in biomedical applications [145]. Through the application of machine learning (ML) and deep learning (DL) techniques, AI facilitates the rapid screening and fine-tuning of MOF structures by analyzing extensive datasets to reveal intricate correlations between structure, properties, and functionality [146]. This accelerates the identification of MOFs with desirable characteristics such as large surface area, biocompatibility, and targeted binding capabilities for specific biomarkers [147]. Lu et al. demonstrated an ML-based approach using carbonized MOFs (C-ZIF-67) for dual purposes: electrochemical sensing and energy storage [148]. They developed an artificial neural network (ANN) model, integrated with theoretical calculations, to predict supercapacitor performance and enhance the electrochemical detection of niclosamide (NA). Their system yielded a 196.6-fold enhancement in signal response compared to a bare glassy carbon electrode, achieving an ultra-low detection limit of 0.3 nM (Figure 11). Furthermore, the supercapacitor demonstrated a high specific capacitance of 336.67 F/g at 2 A/g, confirming the value of integrating ML and theoretical modeling in creating multifunctional electrode materials.

In microfluidic systems, AI further contributes by optimizing chip design, fluid dynamics, and sensor integration, leading to improved analyte capture and signal transduction [149]. AI-enabled platforms also facilitate real-time signal processing, data interpretation, and adaptive calibration, significantly enhancing the sensitivity and reliability of diagnostic outputs [150]. This synergy facilitates the development of smart, automated systems suitable for high-throughput screening and point-of-care applications [151,152].

In the context of single-cell assays, MOFs provide a highly tunable, high-surface-area environment that enhances detection of bacterial viability markers [153]. AI algorithms can analyze MOF-generated optical or electrochemical signals to rapidly distinguish live from dead bacteria, quantify pathogen loads, and predict antimicrobial responses.

Furthermore, AI and ML are revolutionizing MOF-based sensor development through predictive modeling and accelerated material discovery [154]. These tools enable the identification of optimal MOFs for specific analytes by correlating material properties with experimental outcomes [155]. As real-time sensing becomes increasingly important in clinical diagnostics, AI-driven systems offer adaptive learning capabilities that can evolve with patient-specific data, paving the way for intelligent, self-regulating MOF-microfluidic devices for personalized health care [156,157].

#### 5.2.3. MOF-Based Microfluidic Hybrids Manufactured Through 3D Printing

Three-dimensional printing has emerged as a transformative technique in fabricating MOF-integrated microfluidic devices, offering unmatched design flexibility and spatial control [158]. This additive manufacturing approach allows precise embedding of MOFs within defined microchannel architectures, enabling the creation of complex, compartmentalized, and multifunctional environments [159]. Such integration is crucial for simulating organ-like systems (e.g., organ-on-a-chip), achieving spatially resolved sensing, or delivering site-specific treatments [160]. Additionally, the rapid prototyping enabled by 3D printing accelerates device iteration and scalability, paving the way for translational research and potential commercialization of user-specific biomedical microdevices.

## 6. Conclusions and Outlooks

Metal–organic framework (MOF)-based microfluidic chips have emerged as a transformative platform for biomedical applications, offering unparalleled advantages in sensitivity, selectivity, and multifunctionality. This review has highlighted key advancements in MOF material design, integration strategies, and applications in biosensing, drug delivery, diagnostics, and tissue engineering, while also addressing critical challenges such as stability, biocompatibility, and scalability. Looking ahead, the field holds immense potential through the development of smart, stimuli-responsive MOFs, AI-assisted design for optimized structures, and hybrid systems combining MOFs with other nanomaterials for enhanced functionality. Additionally, efforts in standardization, regulatory compliance, and scalable fabrication will be crucial for translating these technologies into commercial and clinical use. By fostering interdisciplinary collaboration among chemists, engineers, and clinicians, MOF-based microfluidics can bridge the gap between lab-scale innovation and real-world biomedical solutions, paving the way for next-generation point-of-care diagnostics, personalized medicine, and advanced therapeutic platforms. Continued progress in material science, microfluidic engineering, and AI-driven optimization will be essential to unlock the full potential of these systems in revolutionizing health care.

## Abbreviations

MOF	Metal–organic framework
COFs	covalent organic frameworks
ZIF8	Zeolitic Imidazolate Framework-8
MIL-88A	Materials of Institute Lavoisier-88A
UiO-66(Zr)	University of Oslo-66 (Zirconium)
LOD	limit of detection
GOx	glucose oxidase
HRP	horseradish peroxidase
PDA	polydopamine
PEI	polyethyleneimine
GCE	glassy carbon electrode
CA15-3	tumor marker
DPV	differential pulse voltammetry
nano-Yb-PVDC-3	nano-Yb-phenylenevinylenedicarboxylate-3
NIR	near-infrared
FeTPt	MOF incorporated meso-tetra(4-carboxyphenyl)porphine
CCM	cancer cell membrane
IVIS	in vivo imaging system
ICG	indocyanine green
BDC	benzene 1,4-dicarboxylic acid
aPDT	antimicrobial photodynamic therapy
RB	Rose Bengal
CTC	circulating tumor cells
FRET	fluorescent resonance energy transfer)
NporMOF(Fe)	nano-metalloporphyrinic
NO	nitic oxide
H_2_O_2_	hydrogen peroxide
BOC	bone-on-a-chip
POCT	point-of-care testing
LBL	layer-by-layer
PICs	photonic integrated circuits
BSA	bovine serum albumin
TA	tannic acid
MRRs	micro-ring resonators
MZIs	Mach–Zehnder interferometers
LPE	liquid-phase epitaxy
BDC-NH_2_	2-aminoterephthalic acid
MEAs	metal microelectrode arrays
HOFs	hydrogen-bonded organic frameworks
AuNPs	gold nanoparticles
VOCs	volatile organic compound
CbAgo	clostridium butyricum argonaute
PDMS	polydimethylsiloxane
L-Trp	L-tryptophan
TAC	total antioxidant capacity
TMB	3,3′,5,5′-Tetramethylbenzidine
VSA	virtual sensor array
QCM	quartz crystal microbalance
